# Genistein improves inflammatory response and colonic function through NF-κB signal in DSS-induced colonic injury

**DOI:** 10.18632/oncotarget.18219

**Published:** 2017-05-26

**Authors:** Rui Zhang, Jian Xu, Jian Zhao, Yuzhe Chen

**Affiliations:** ^1^ Department of Colorectal Surgery, Cancer Hospital of China Medical University, Liaoning Cancer Hospital & Institute, Shenyang 110042, Liaoning Province, P. R. China

**Keywords:** genistein, inflammation, barrier, NF-κB, mice

## Abstract

This study aimed to investigate the protective potential of genistein in dextran sulfate sodium (DSS)-induced colonic injury *in vitro* and *in vivo* models. The results showed that DSS exposure caused growth suppression, colonic injury, inflammation, and barrier dysfunction in mice. Dietary genistein alleviated DSS-caused colonic injury via reducing colonic weight, rectal bleeding, and diarrhea ratio. Meanwhile, genistein reduced colonic inflammatory response via downregulating cytokines expression and improved colonic permeability and barrier in DSS-challenged mice. In Caco-2 cells, genistein improved cell viability and cellular permeability and inhibited DSS-induced activation of TLR4/NF-κB signal. In conclusion, genistein alleviated DSS-caused colonic injury, inflammation, and gut dysfunction, which might be associated with the TLR4/NF-κB signal.

## INTRODUCTION

Various dietary nutrients have been identified as potential adjuvants to prevent different chronic diseases and ameliorate pharmacological therapies, such as inflammatory bowel disease (IBD). Genistein, a soy derived isoflavanoid compound serves as a potent agent in both prophylaxis and treatment of cancer and various other chronic diseases [1]. Currently, studies about genistein mainly focuses on its preventative and therapeutic effects for cancers [2, 3]. Genistein acts as an anti-cancer agent mainly by mediating apoptosis process, cell cycle, and angiogenesis and inhibiting metastasis. Meanwhile, genistein also has been showed anti-inflammatory effect in various models [4]. For example, genistein at physiological concentrations (0.1 μM-5 μM) inhibits tumor necrosis factor α (TNF-α)-induced endothelial inflammatory response and vascular inflammation in C57BL/6 mice via mediating the protein kinase pathway A [5]. Meanwhile, various reports have shown that genistein inactivates nuclear factor-kappa B (NF-κB) signal [6, 7], which is widely associated with the development and pathological mechanism of inflammatory diseases [8, 9].

In this study, the protective effect of genistein on dextran sulfate sodium (DSS)-induced colonic injury and inflammation was investigated in mice and Caco-2 cells. The results concluded that genistein alleviated DSS-caused colonic injury, inflammation, and gut dysfunction, which might be associated with the TLR4/NF-κB signal.

## RESULTS

### Growth performance

**Figure 1:**
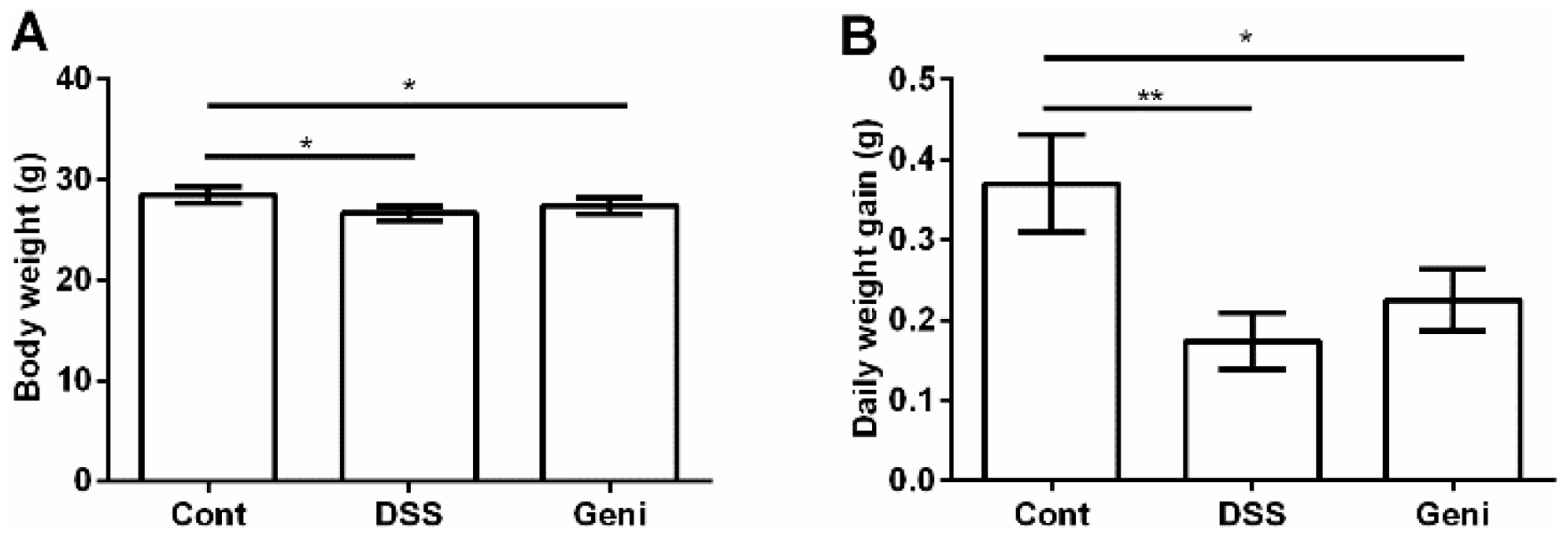
Effect of genistein on final body weight **(A)** and average daily gain **(B).** Data are expressed as the mean ± standard error of the mean (n=10). * Means the difference was significant (P<0.05).

As shown at Figure 1, DSS exposure markedly inhibited growth in mice via reducing body weight and average daily weight gain (P<0.05). Dietary supplementation with genistein tended to alleviate DSS-caused growth suppression, but the difference was insignificant (P>0.05).

### Colonic injury

**Figure 2:**
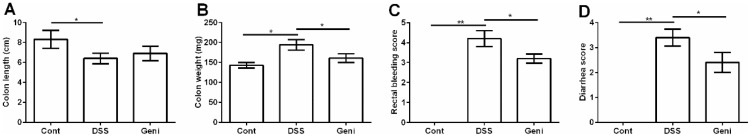
Genistein alleviated DSS-induced colonic injury in mice Data are expressed as the mean ± standard error of the mean (n=10). * Means the difference was significant (P<0.05).

DSS exposure significantly caused colonic injury evidenced by the decreased colonic length (Figure 2A) and increased colonic weight (Figure 2B), rectal bleeding (Figure 2C), and diarrhea ratio (Figure 2D) (P<0.05). Compared with the DSS group, colonic weight, rectal bleeding score, and diarrhea score were significantly lower in DSS+Geni group (P<0.05), suggesting a protective role of genistein in DSS-induced colonic injury.

### Colonic inflammation

Interleukins (IL-1β, IL-6, IL-10, IL-17), TNF-α, and interferon gamma (IFN-γ) were determined to evaluate colonic inflammatory response after DSS exposure via RT-PCR (Table 1). DSS-challenged mice showed marked upregulation of IL-1β, IL-17, TNF-α, and IFN-γ (P<0.05), while genistein treatment inhibited colonic production of IL-1β and IFN-γ (P<0.05).

**Table 1:** Genistein alleviated DSS-induced colonic inflammation

Item	Cont	DSS	Geni
IL-1β	1.00 ± 0.04	1.49 ± 0.10*	1.22 ± 0.16#
IL-6	1.00 ± 0.15	1.27 ± 0.14	1.26 ± 0.15
IL-10	1.00 ± 0.09	0.95 ± 0.09	1.23 ± 0.21
IL-17	1.00 ± 0.17	1.32 ± 0.13*	1.14 ± 0.26
TNF-α	1.00± 0.07	1.47 ± 0.16*	1.41 ± 0.02
IFN-γ	1.00 ± 0.06	1.56 ± 0.13*	1.31 ± 0.14#

Note: * means the difference was significant compared with the control group (P<0.05); # means the difference was significant compared with the DSS group (P<0.05).

### Colonic permeability and barrier

Serum lipopolysaccharide (LPS) and diamine oxidase (DAO) activity have been widely used to evaluate gut permeability. In this study, we found that DSS markedly increased serum LPS abundance compared with the control group (P<0.05) (Table 2), while serum LPS in the DSS+Geni group was significantly lower than that in DSS group (P<0.05) (Table 2). Serum DAO activity in this study failed to be affected after DSS and genistein administration.

**Table 2:** Genistein alleviated DSS-induced colonic dysfunction

Item	Cont	DSS	Geni
LPS (ng/ml)	52.75±4.53	75.47±4.56*	63.82±2.64#
DAO (U/ml)	102.19±6.74	93.37±4.96	94.53±3.28

Note: * means the difference was significant compared with the control group (P<0.05); # means the difference was significant compared with the DSS group (P<0.05).

Expressions of tight junctions (ZO-1, claudin1, cluadin2, and occludin) were further investigated in the colon via RT-PCR (Table 3). The results showed that DSS downregulated ZO-1, Claudin2, and occludin expressions compared with the control group (P<0.05). Genistein administration markedly enhanced colonic ZO-1 and occludin mRNA abundances after DSS exposure in mice (P<0.05).

**Table 3:** Genistein enhanced colonic expressions of tight junctions in DSS-challenged mice

Item	Cont	DSS	Geni
ZO-1	1.00 ± 0.11	0.73 ± 0.11*	0.89 ± 0.13#
Claudin1	1.00 ± 0.15	1.18 ± 0.08	1.27 ± 0.19
Claudin2	1.00 ± 0.05	0.78 ± 0.09*	0.83 ± 0.06
Occludin	1.00 ± 0.13	0.65 ± 0.04*	0.86 ± 0.05#

Note: * means the difference was significant compared with the control group (P<0.05); # means the difference was significant compared with the DSS group (P<0.05).

### Cell viability and cellular permeability in Caco-2 cells

**Figure 3:**
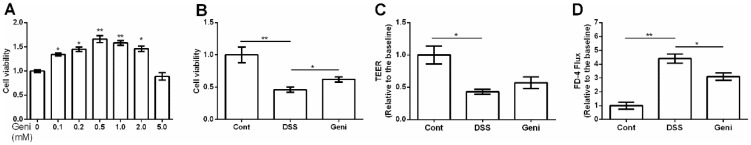
Effect of genistein on cell viability and permeability in Caco-2 cells Data are expressed as the mean ± standard error of the mean (n=3 or 6). * Means the difference was significant (P<0.05).

0.1-2 mM genistein markedly enhanced cell viability (Figure 3A) and 0.5 mM was used for following analysis. We found that 0.5 mM genistein treatment alleviated the decreased cell viability caused by DSS exposure (P<0.05) (Figure 3B).

Trans-epithelial electrical resistance (TEER) was measured in the Caco-2 monolayers and the results showed that DSS markedly reduced TEER (P<0.05) (Figure 3C). Although genistein tended to enhance TEER in the Caco-2 monolayers after DSS exposure, the difference was insignificant TEER (P>0.05) (Figure 3C).

In this study, paracellular marker FD-4 (FITC-Dextran 4 kDa) flux was also tested in the Caco-2 monolayers after incubation with FD-4, DSS, and genistein. The results concluded that DSS increased cellular permeability evidenced by the increased FD-4 flux (P<0.05) (Figure 3D). Meanwhile, genistein treatment markedly reduced FD-4 flux compared with DSS group (P<0.05) (Figure 3D).

### TLR4/NF-κB signal in Caco-2 cells

**Figure 4:**
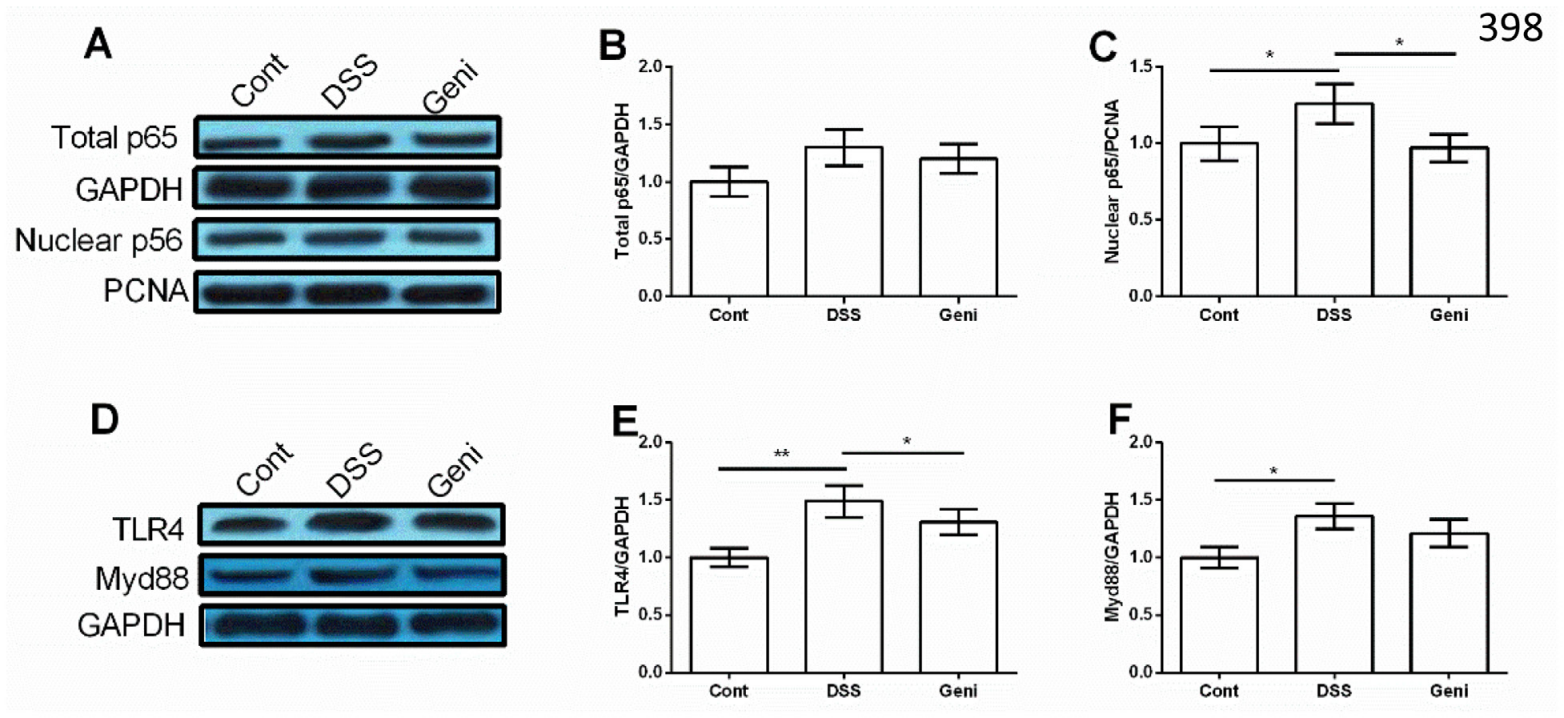
Genistein inhibited DSS-induced activation of TLR4/NF-κB signal in Caco-2 cells (n=3). * Means the difference was significant (P<0.05).

Although we failed to notice any significant difference in total NF-κBp65 in Caco-2 cells (P>0.05) (Figure 4A and 4B), DSS treatment markedly enhanced nuclear NF-κBp65 abundance and genistein reduced nuclear NF-κBp65 compared with the DSS group (P<0.05) (Figure 4A and 4C).

TLR4 and Myd88, two upstream proteins of NF-κB signal, were markedly activated in DSS group (P<0.05) (Figure 4D-4F) and genistein treatment inhibited TLR4 expression in Caco-2 cells after DSS exposure (P<0.05) (Figure 4E).

## DISCUSSION

Genistein (4', 5, 7-trihydroxyisoflavone), one of the major soy isoflavones, has been identified a wide variety of biological activities, such as regulating cell proliferation and cell cycle, induction of apoptosis, inhibition of NF-κB activation, and anti-inflammatory and antioxidant effects [10]. In a rat model of IBD, a low dose of fermented soy germ alleviated TNBS (2,4,6-trinitrobenzene sulphonic acid)-induced colonic injury [11]. Similarly, the present data showed that dietary genistein attenuated colonic injury via improving colonic weight, rectal bleeding, and diarrhea ratio, suggesting a protective role of genistein in DSS-induced colonic injury in mice.

DSS has been widely used to induce colonic inflammation in animals [12, 13]. In this study, DSS administration for 7 days markedly caused colonic inflammatory response by upregulating colonic expression of pro-inflammatory cytokines (IL-1β, IL-17, TNF-α, and IFN-γ). Dietary supplementation with genistein inhibited the overexpression of IL-1β and IFN-γ, suggesting an anti-inflammatory functions in DSS-induced colonic inflammation. Genistein also has been showed anti-inflammatory function in other models. For example, genistein decreased the secretion of IL-1β, IL-6, and IL-8 in TNF-α-stimulated MH7A cells and the mechanism involved in denosine monophosphate-activated protein kinase (AMPK) and NF-κB signals [14]. In streptozotocin-induced diabetic rats, administration of genistein resulted in a marked decrease in C-reactive protein, TNF-α, transforming growth factor (TGF-β1), and oxidative stress [15]. Oxidative stress also involves in the development of IBD and colitis-associated colorectal cancer and antioxidant agents as pharmacological targets for anti-IBD drugs [16, 17]. Although we failed to investigate colonic antioxidant status in this study, genistein has been widely demonstrated to exhibit antioxidant function in inflammatory diseases [15, 18].

Intestinal dysfunction with increased permeability and downregulated tight junctions plays an important role in the pathogenesis of IBD and other inflammatory diseases [19, 20]. In this study, we found that DSS increased permeability (serum LPS) and downregulated tight junctions expression, while genistein markedly alleviated DSS-induced colonic dysfunction in mice. The *in vivo* data further confirmed that genistein improved cellular permeability in Caco-2 cells. Genistein protects barrier function against oxidative stress, acetaldehyde, enteric bacteria and inflammatory cytokines and blocks the tyrosine phosphorylation of the tight junctions induced by oxidative stress and acetaldehyde, which results in the disassembly of the proteins from the junctional complex [21].

Activation of NF-κB signaling pathways is closely associated with the development of IBD [22–24]. Genistein has been shown to inhibit the activity of NF-κB signaling pathways [25], which might be a potential agent to protect against DSS-induced colonic inflammation. In this study, genistein reduced nuclear NF-κBp65 compared with the DSS group in Caco-2 cells. Meanwhile, TLR4, an upstream protein of NF-κB signal, was markedly inhibited in Caco-2 cells after DSS and genistein exposure.

## MATERIALS AND METHODS

### Experimental design

30 female BALB/C mice (19.41 ± 1.66 g) were housed in polycarbonate cages in a room with controlled temperature (25 ± 3 °C), humidity (50 ± 5%) and a 12 hour cycle of light and dark and randomly divided into three groups: a control group (Cont, n = 10), a DSS group (DSS, n = 10) in which mice received 3% DSS (KAYON Bio. Technology Co. Ltd) instead for tap water for 7 days to establish IBD model [26], and a DSS plus genistein group (DSS+Geni). Genistetin was administrated via adding 600 mg genistein/kg diet in the feeding diet [27].

Mice were weighed and sacrificed at day 8. Colonic length and weight were recorded, and then frozen in liquid nitrogen for further analysis. This study was conducted according to the guidelines of the Declaration of Helsinki and all procedures involving animal subjects were approved by the animal welfare committee of Cancer Hospital of China Medical University, Liaoning Cancer Hospital.

### Clinical evaluation of DSS colitis

At day 8, rectal bleeding and diarrhea in each mouse were recorded. Blood in the stool was tested via haemoccult tests (Beckman Coulter), and was given a score from 0 to 4, defined as follows: 0 for no blood; 2 for positive haemoccult; and 4 for gross bleeding. The severity of diarrhea was given a score from 0 to 4, defined as follows: 0 for well-formed pellets; 2 for pasty and semiformed stools; and 4 for liquid stools [28].

### Serum LPS and DAO

Blood samples were harvested via orbital blood sampling and serum samples were separated from blood by centrifugation at 3,500 × g for 15 min under 4 °C. Serum LPS level and DAO activity were measured using assay kits in accordance with the manufacturer’s instructions (BiovisionInc., USA)

### Real-time PCR

Total RNA from colonic samples was isolated with TRIZOL regent (Invitrogen, USA) and reverse transcribed into the first strand (cDNA) using DNase I, oligo (dT) 20 and Superscript II reverse transcriptase (Invitrogen, USA). Primers were designed with Primer 5.0 according to the gene sequence of mouse to produce an amplification product (Table 4). β-actin was chosen as the house-keeping gene to normalize target gene levels. The PCR cycling condition was 36 cycles at 94°C for 40 sec, 60 °C for 30 sec and 72°C for 35 sec. The relative expression was expressed as a ratio of the target gene to the control gene using the formula 2-^(ΔΔCt)^, where ΔΔCt=(Ct_Target_-Ct_β-actin_)treatment-(Ct_Target_-Ct_β-actin_)_control_. Relative expression was normalized and expressed as a ratio to the expression in the control group.

**Table 4:** Primers used for RT-PCR in this study

Genes	Sequence ID	Nucleotide sequence of primers (5′–3′)	bp
β-Actin	NM_007393.5	F: CCACCATGTACCCAGGCATT R: AGGGTGTAAAACGCAGCTCA	253
IL-1β	NM_008361.4	F: TGCCACCTTTTGACAGTGATG R: AAGGTCCACGGGAAAGACAC	220
IL-6	NM_031168.2	F: CCCCAATTTCCAATGCTCTCC R: CGCACTAGGTTTGCCGAGTA	141
IL-10	NM_010548.2	F: TAAGGCTGGCCACACTTGAG R: GTTTTCAGGGATGAAGCGGC	209
IL-17	NM_010552.3	F: GCTGACCCCTAAGAAACCCC R: GAAGCAGTTTGGGACCCCTT	162
TNF-α	NM_013693.3	F: ATGGCCTCCCTCTCATCAGT R:TTTGCTACGACGTGGGCTAC	97
IFN-γ	NM_008337.4	F: CGGCACAGTCATTGAAAGCC R: TGCATCCTTTTTCGCCTTGC	268
ZO-1	NM_009386.2	F: GCCTTGAACTTTGACCTCTGC R: GAAATCGTGCTGATGTGCCA	243
Claudin1	NM_016674.4	F: GGCTTCTCTGGGATGGATCG R: CCCCAGCAGGATGCCAATTA	235
Claudin2	NM_016675.4	F: ATGCCTTCTTGAGCCTGCTT R: AAGGCCTAGGATGTAGCCCA	218
Occludin	NM_008756.2	F: CCGGCCGCCAAGGTTC R: GCTGATGTCACTGGTCACCTA	78

F: forward; R: reverse; IL: interleukin; TNF: Tumor necrosis factor; IFN-γ: interferon gamma.

### Cell lines and cell culture

Human epithelial Caco-2 cells (ATCC, Wuhan Procell, China) were grown in Dulbecco’s modified Eagle medium (DMEM)/F12 supplemented with 10% FBS (HyClone, Logan, UT) and 50 U/mL penicillin–streptomycin and maintained at 37 °C in a humidified chamber of 5% CO_2_. Confluent cells (85–90%) were incubated with different concentrations of genistein and 2% DSS for 4 days to establish inflammatory model [29].

### Cell viability

Cell viability was measured by the CKK-8 assay (Sigma–Aldrich). Briefly, cells dispersed evenly in medium were seeded in a 96-well plate with a density of 1x10^4^ cells/well. Next day, cells were treated with genistein and DSS for 4 days. After incubation, CKK-8 solution was added to each well, followed by a 2 h incubation. The optical density (OD) in 570 nm was measured by a BioTek multilabel counter.

## TEER

Caco-2 cells were grown in a 12-well Trans-well system and the changes of TEER were determined using an epithelial voltohmmeter ERS-2 (Merck Millipore, USA). When the filter-grown Caco-2 monolayers reached epithelial resistance of at least 500 Ω cm^2^, the cells were incubated with genistein and DSS treatment. Electrical resistance was measured until similar values were recorded on three consecutive measurements. Values were corrected for background resistance due to the membrane insert and calculated as Ω cm^2^.

### FD-4 flux

Paracellular permeability was estimated via FD-4 flux. Briefly, Caco-2 cells were seeded in a 12-well Trans-well system to reach monolayers. After treatment with genistein and DSS, cells were incubated in the upper chamber with Hank’s balanced salt solution for 2 h, which contains 1 mg/mL FD-4 solution. FD-4 signal was determined via Synergy H2 microplate reader (Biotek Instruments, USA).

### Western blot

Total proteins and nuclear proteins from colonic samples were extracted with using protein extraction reagents (Thermo Fisher Scientific Inc., USA) and the concentration was tested using BCA protein assay (Sigma-Aldrich, USA). Proteins (30-50 μg) were separated by SDS–polyacrylamide gel electrophoresis and electrophoretically transferred to a polyvinylidene difluoride (PVDF) membrane (BioRad, Hercules, CA, USA). Membranes were blocked and then incubated with the following primary antibodies: anti-NF-kBp65 (ab16502), anti-TLR4 antibody (ab13556), anti-MyD88 antibody (ab2068), anti-PCNA antibody (ab18197), and anti-beta Actin antibody (ab8227). After primary antibody incubation, membranes were washed, incubated with alkaline phosphatase-conjugated anti-mouse or anti-rabbit IgG antibodies (Promega, Madison, WI, USA), and quantified and digitally analyzed using the image J program (NIH).

### Statistical analysis

All data were analyzed by IBM SPSS 21.0 software. Difference was tested by student’s t test. Data are expressed as the mean ± SEN. P < 0.05 means the difference is significant.

## References

[1] Ganai AA, Farooqi H (2015). Bioactivity of genistein: a review of *in vitro* and *in vivo* studies. Biomed Pharmacother.

[2] Spagnuolo C, Russo GL, Orhan IE, Habtemariam S, Daglia M, Sureda A, Nabavi SF, Devi KP, Loizzo MR, Tundis R, Nabavi SM (2015). Genistein and cancer: current status, challenges, and future directions. Adv Nutr.

[3] Pavese JM, Krishna SN, Bergan RC (2014). Genistein inhibits human prostate cancer cell detachment, invasion, and metastasis. Am J Clin Nutr.

[4] Nagaraju GP, Zafar SF, El-Rayes BF (2013). Pleiotropic effects of genistein in metabolic, inflammatory, and malignant diseases. Nutr Rev.

[5] Jia Z, Babu PV, Si H, Nallasamy P, Zhu H, Zhen W, Misra HP, Li Y, Liu D (2013). Genistein inhibits TNF-alpha-induced endothelial inflammation through the protein kinase pathway A and improves vascular inflammation in C57BL/6 mice. Int J Cardiol.

[6] Palanisamy N, Kannappan S, Anuradha CV (2011). Genistein modulates NF-kappaB-associated renal inflammation, fibrosis and podocyte abnormalities in fructose-fed rats. Eur J Pharmacol.

[7] Khan AQ, Khan R, Rehman MU, Lateef A, Tahir M, Ali F, Sultana S (2012). Soy isoflavones (daidzein & genistein) inhibit 12-O-tetradecanoylphorbol-13-acetate (TPA)-induced cutaneous inflammation via modulation of COX-2 and NF-kappaB in Swiss albino mice. Toxicology.

[8] Pateras I, Giaginis C, Tsigris C, Patsouris E, Theocharis S (2014). NF-kappa B signaling at the crossroads of inflammation and atherogenesis: searching for new therapeutic links. Expert Opin Ther Targets.

[9] Nguyen DP, Li JY, Yadav SS, Tewari AK (2014). Recent insights into NF-kappa B signalling pathways and the link between inflammation and prostate cancer. BJU Int.

[10] Yang Z, Kulkarni K, Zhu W, Hu M (2012). Bioavailability and pharmacokinetics of genistein: mechanistic studies on its ADME. Anticancer Agents Med Chem.

[11] Moussa L, Bezirard V, Salvador-Cartier C, Bacquie V, Lencina C, Leveque M, Braniste V, Menard S, Theodorou V, Houdeau E (2012). A low dose of fermented soy germ alleviates gut barrier injury, hyperalgesia and faecal protease activity in a rat model of inflammatory bowel disease. PLos One.

[12] Martinez Gomez JM, Chen L, Schwarz H, Karrasch T (2013). CD137 facilitates the resolution of acute DSS-induced colonic inflammation in mice. PLoS One.

[13] Shi C, Liang Y, Yang J, Xia Y, Chen H, Han H, Yang Y, Wu W, Gao R, Qin H (2013). MicroRNA-21 knockout improve the survival rate in DSS induced fatal colitis through protecting against inflammation and tissue injury. PLoS One.

[14] Li JC, Li J, Yue Y, Hu YP, Cheng WX, Liu RX, Pan XH, Zhang P (2014). Genistein suppresses tumor necrosis factor alpha-induced inflammation via modulating reactive oxygen species/Akt/nuclear factor kappa B and adenosine monophosphate-activated protein kinase signal pathways in human synoviocyte MH7A cells. Drug Des Devel Ther.

[15] Gupta SK, Dongare S, Mathur R, Mohanty IR, Srivastava S, Mathur S, Nag TC (2015). Genistein ameliorates cardiac inflammation and oxidative stress in streptozotocin-induced diabetic cardiomyopathy in rats. Mol Cell Biochem.

[16] Pereira C, Gracio D, Teixeira JP, Magro F (2015). Oxidative stress and DNA damage: implications in inflammatory bowel disease. Inflamm Bowel Dis.

[17] Piechota-Polanczyk A, Fichna J (2014). Review article: the role of oxidative stress in pathogenesis and treatment of inflammatory bowel diseases. Naunyn Schmiedebergs Arch Pharmacol.

[18] Incir S, Bolayirli IM, Inan O, Aydin MS, Bilgin IA, Sayan I, Esrefoglu M, Seven A (2016). The effects of genistein supplementation on fructose induced insulin resistance, oxidative stress and inflammation. Life Sci.

[19] Howell K, Yan F, Tokich A, Ng K (2015). Iron sequestration is not the main mechanism in the inhibition of Staphylococcus aureus growth by cranberry phytochemicals. Integr Food Nutr Metab.

[20] Ande SR, Nguyen KH, Nyomba BL, Mishra S (2016). Prohibitin in adipose and immune functions. Trends Endocrinol Metab.

[21] Suzuki T, Hara H (2011). Role of flavonoids in intestinal tight junction regulation. J Nutr Biochem.

[22] Luettig J, Rosenthal R, Lee IM, Krug SM, Schulzke JD (2016). The ginger component 6-shogaol prevents TNF-alpha-induced barrier loss via inhibition of PI3K/Akt and NF-kappaB signaling. Mol Nutr Food Res.

[23] Giner E, Recio MC, Rios JL, Cerda-Nicolas JM, Giner RM (2016). Chemopreventive effect of oleuropein in colitis-associated colorectal cancer in c57bl/6 mice. Mol Nutr Food Res.

[24] Kim H, Banerjee N, Ivanov I, Pfent CM, Prudhomme KR, Bisson WH, Dashwood RH, Talcott ST, Mertens-Talcott SU (2016). Comparison of anti-inflammatory mechanisms of mango (Mangifera Indica L.) and pomegranate (Punica Granatum L.) in a preclinical model of colitis. Mol Nutr Food Res.

[25] Du Q, Wang Y, Liu C, Wang H, Fan H, Li Y, Wang J, Zhang X, Lu J, Ji H, Hu R (2016). Chemopreventive activity of GEN-27, a genistein derivative, in colitis-associated cancer is mediated by p65-CDX2-beta-catenin axis. Oncotarget.

[26] Marcon R, Claudino RF, Dutra RC, Bento AF, Schmidt EC, Bouzon ZL, Sordi R, Morais RL, Pesquero JB, Calixto JB (2013). Exacerbation of DSS-induced colitis in mice lacking kinin B(1) receptors through compensatory up-regulation of kinin B(2) receptors: the role of tight junctions and intestinal homeostasis. Br J Pharmacol.

[27] Odle B, Dennison N, Al-Nakkash L, Broderick TL, Plochocki JH (2017). Genistein treatment improves fracture resistance in obese diabetic mice. BMC Endocr Disord.

[28] Vlantis K, Polykratis A, Welz PS, van Loo G, Pasparakis M, Wullaert A (2016). TLR-independent anti-inflammatory function of intestinal epithelial TRAF6 signalling prevents DSS-induced colitis in mice. Gut.

[29] Nighot P, Young K, Nighot M, Rawat M, Sung EJ, Maharshak N, Plevy SE, Ma T, Blikslager A (2013). Chloride channel ClC-2 is a key factor in the development of DSS-induced murine colitis. Inflamm Bowel Dis.

